# Supplementary material for: Phage display and selection of lanthipeptides on the carboxy-terminus of the gene-3 minor coat protein
Source: Nat Commun. 2017 Nov 15;8:1500. doi: 10.1038/s41467-017-01413-7 (PMC5686179; doi:10.1038/s41467-017-01413-7)
Supplement: Supplementary file 1 — Supplementary Information [file 41467_2017_1413_MOESM1_ESM.pdf]

Supplementary Figure 1

a

Reporter peptides (cycle size)	Core sequence
Nis-6 and Proc-6 (i, i+7)	ASWIEGRECN
Nis-9 and Proc-9 (i, i+10)	ASWAAIEGRAECN
Nis-12 and Proc-12 (i, i+13)	ASWAAAIEGRAAAECN
Nis-16 and Proc-16 (i, i+17)	ASWAAGAAIEGRAAGAAECN

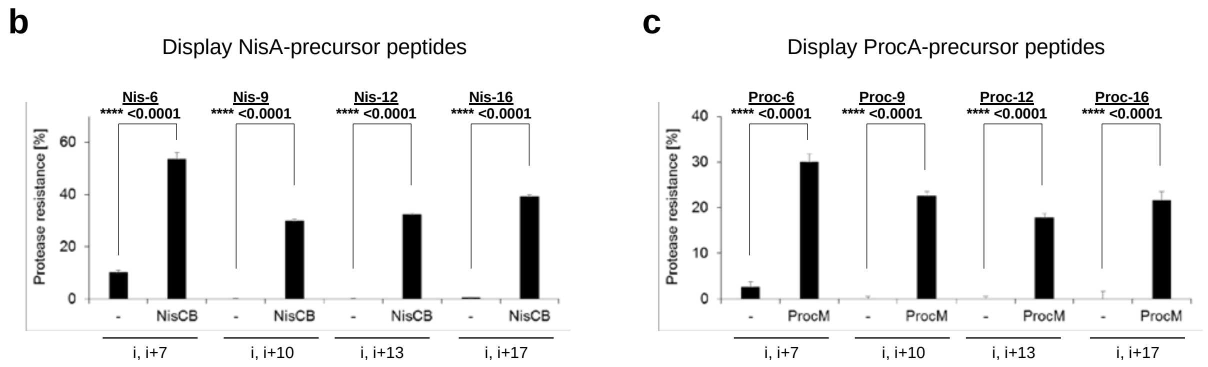

Supplementary Figure 1 Display of reporter peptides with increased monocycle size on the C-terminus of phage pIII. (**a**) Cycle size and sequence of tested core peptides. Residues involved in thioether formation are colored, the FXa-site is underlined. (**b**) ELISA-based FXa-cleavage reporter assay performed on phage particles displaying indicated peptide sequences (full sequences in Supplementary Table 3) fused to the NisA-leader and produced with or without NisBC co-expression. The protease resistance relative to untreated (no FXa) samples was calculated and data representing mean ± s.d. of three independent phage preparations analyzed in duplicate is shown (unpaired, two-tailed *t*-test). (**c**) As in (b), but phage displayed model peptides containing ProcA leader sequences (full sequences in Supplementary Table 4) were tested after phage production with or without ProcM co-expression. The experiments shown in panel b and c were repeated twice.

Supplementary Figure 2

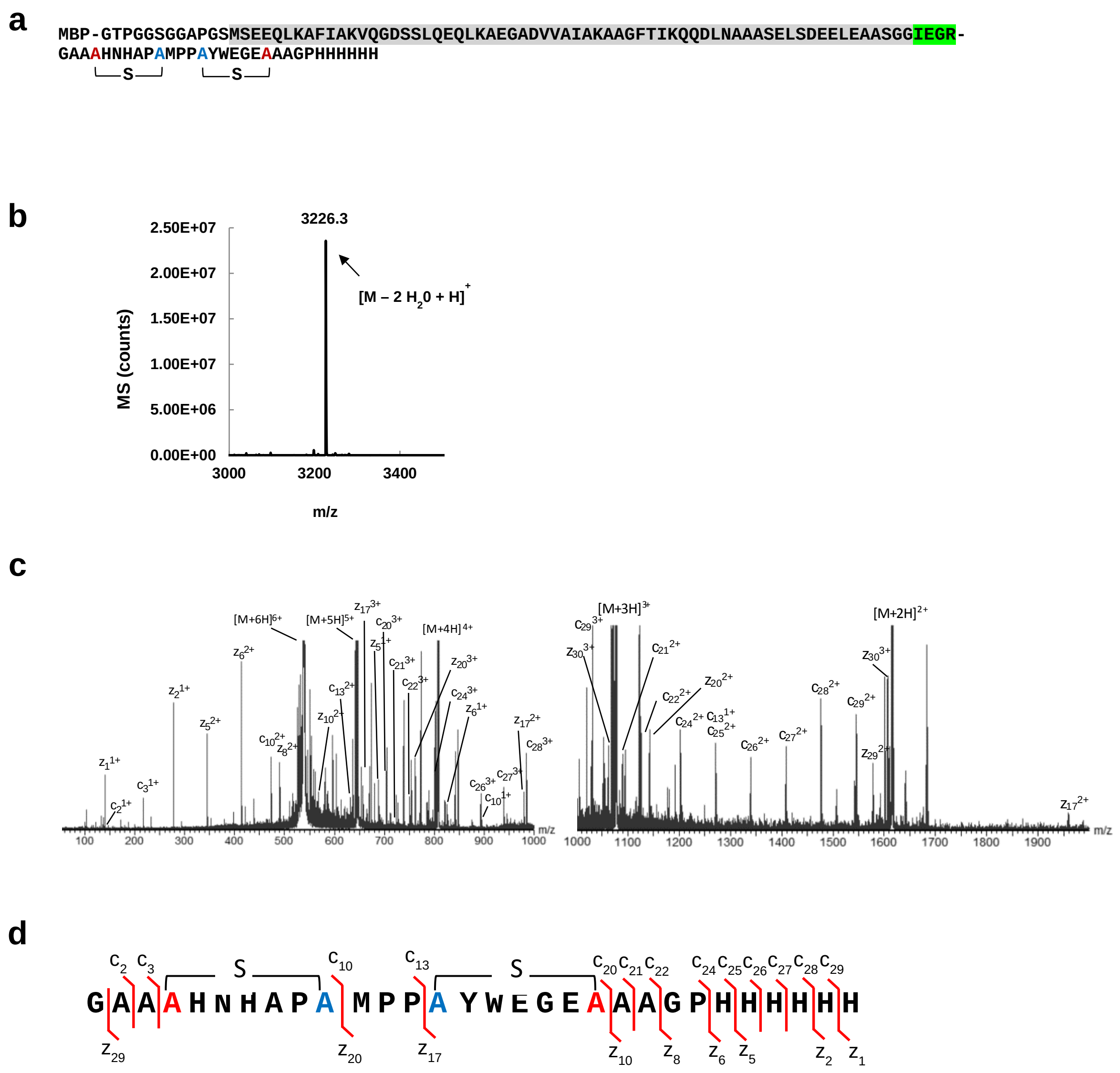

Supplementary Figure 2 Production and analytics of leader peptide-free ProcA2.8 expressed as C-terminal fusion to MBP. (**a**) ProcA2.8 was expressed from plasmid pET21a_MBP_PEP226 as C-terminal fusion to MBP with an FXa-site (green sequence) inserted in the leader (gray sequence)-core junction and with co-expression of ProcM. MBP and the leader peptide were proteolytically removed by FXa treatment and the resulting His_6-tagged core peptide purified by IMAC prior to analysis. (**b**) The ESI mass spectra is shown with the main peak peptide mass reflecting the two fold dehydrated core peptide (indicated by an arrow). (**c**) ETD spectra and assignment of c and z fragments of leader free ProcA2.8 in the ranges of m z^{-1} 100-1000 (left panel) and m z^{-1} 1000-2000 (right panel). (**d**) Sequence of leader free ProcA2.8 and lanthionine structure derived from ETD data. Fragments found under ETD conditions (as detailed in Supplementary Tables 7, 8) are depicted above (c series) and below (z series) the sequence.

Supplementary Figure 3

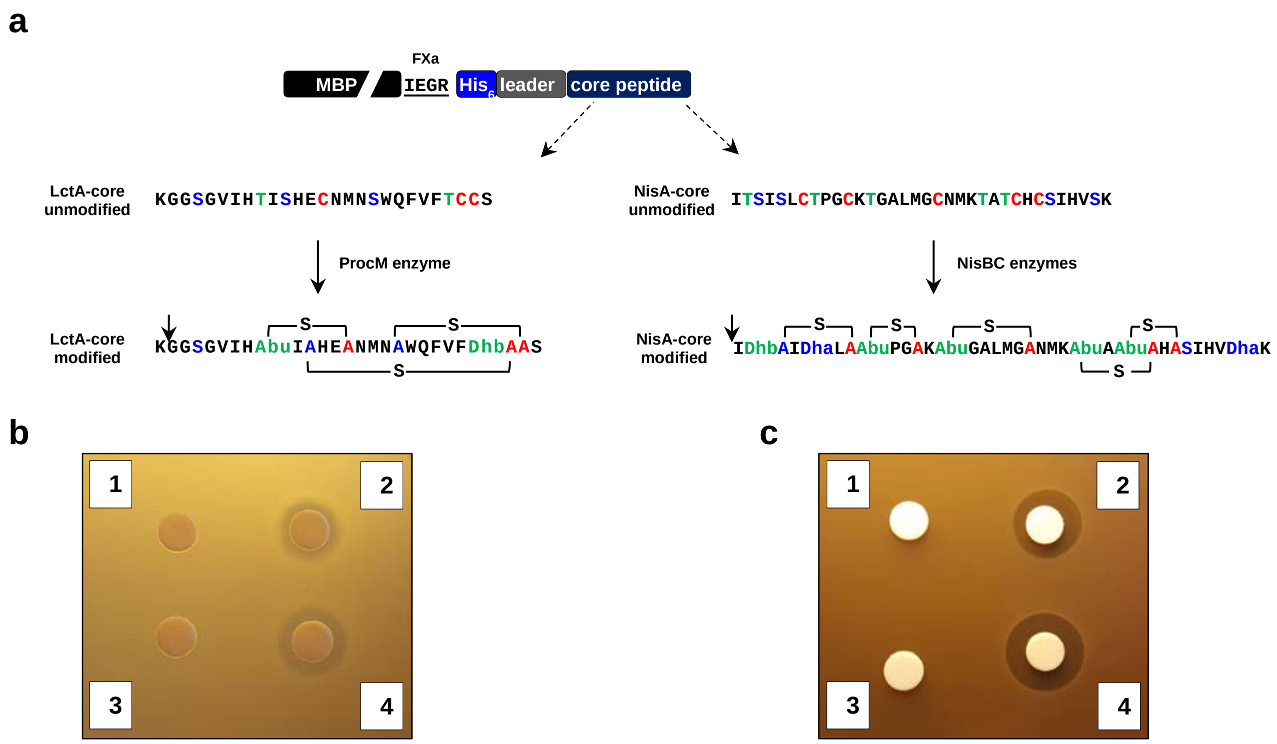

1: PBS + trypsin
2: Authentic lacticin 481
3: MBP_His$_6$_ProcA3.3-LctA + trypsin
4: MBP_His$_6$_ProcA3.3-LctA + ProcM + trypsin

1: PBS + trypsin
2: Authentic Nisin
3: MBP_His$_6$_NisA + trypsin
4: MBP_His$_6$_NisA + NisBC + trypsin

Supplementary Figure 3 Bioactivity assay of lanthipeptides produced as C-terminal fusions to MBP. (**a**) Schematic drawing of the MBP-fusion vector used for expression of a chimeric peptide consisting of the ProcA3.3 leader sequence fused to the core of LctA (left panel) and NisA (right panel; full sequences shown in Supplementary Table 5). The sequence of the unmodified and modified core peptides as reported in the literature are indicated[1]. The arrow indicates a trypsin cleavage site used to release the mature core peptide. (**b**) Growth inhibition of lacticin 481-sensitive *L. lactis cremonis* (HP) by filter-disc spotted peptide solutions. (1) Trypsin treated PBS (negative control); (2) authentic lacticin 481 (positive control); (3) ProcA3.3-LctA produced as C-terminal MBP fusion in *E. coli* without ProcM co-expression and treated with trypsin; (4) ProcA3.3-LctA produced as C-terminal MBP fusion in *E. coli* with ProcM co-expression and treated with trypsin. (**c**) Growth inhibition of nisin-sensitive *L. lactis* NZ9000 by filter-disc spotted peptide solutions. (1) Trypsin treated PBS (negative control); (2) authentic nisin (positive control); (3) NisA produced as C-terminal MBP fusion in *E. coli* without NisBC co-expression and treated with trypsin; (4) NisA produced as C-terminal MBP fusion in *E. coli* with NisBC co-expression and treated with trypsin.

Supplementary Figure 4

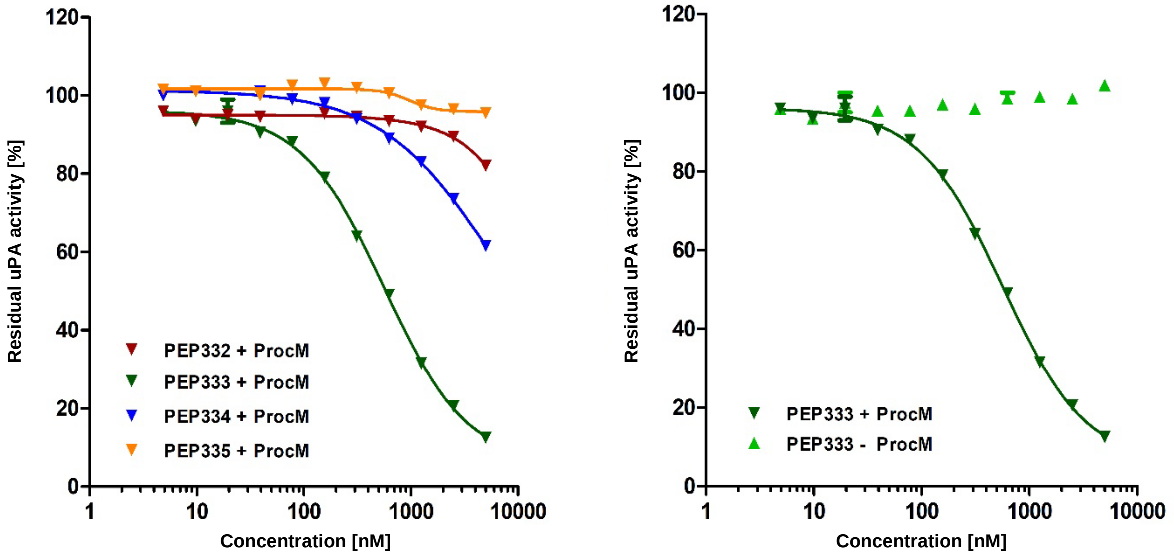

Supplementary Figure 4 Inhibitory activity of anti-uPA lanthipeptides. (**a**) Residual activities of human uPA incubated with ProcM-modified anti-uPA leader-core peptides PEP332 to PEP335 as indicated. (**b**) As in (a), but residual activities of human uPA incubated with PEP333 produced with (+ ProcM) or without (- ProcM) ProcM co-expression. The inhibitory constant *Ki* of PEP333 is 564 nM and was calculated as recently described[2]. The experiment was repeated three times.

Supplementary Figure 5

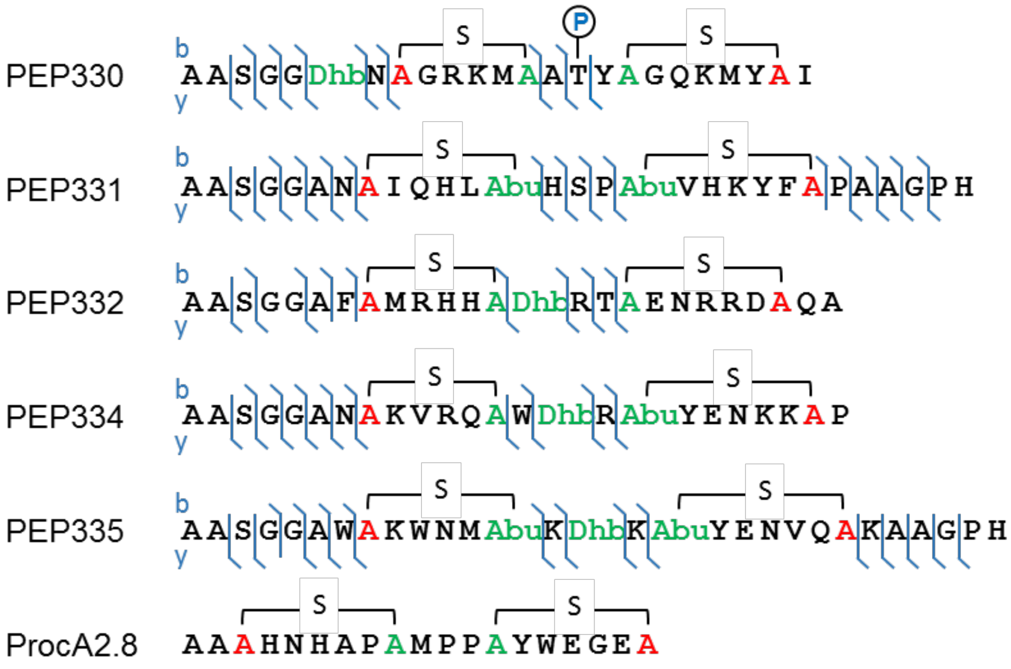

Supplementary Figure 5 Proposed thioether configuration of five ProcM-modified peptides selected by phage display. For comparison the reported structure of the natural substrate ProcA2.8 is shown[3], which inspired the library design used in this study. Thioether-bridges are formed by bridging of a cysteine (A; in red) to a dehydroalanine Dha (resulting in A; in green) or a dehydrobutyrine Dhb (resulting in aminobutyrate Abu). The proposed structures are deduced from the MS data shown in Supplementary Table 9 and the MS/MS data shown in Supplementary Tables 10 – 15. The N-terminal ProcA3.3 leader was partially removed by Glu-C digestion and the linker-His$_6$-tag was partially removed by treatment with carboxypeptidase Y prior to MS/MS analysis. The remaining sequences and their ring structures are shown. The observed phosphorylation (P) of PEP330 was proven to be in between the two lanthionine rings (Supplementary Tables 9, 13 and 14). Phosphorylation is an observed intermediate in the dehydration reaction of Ser and Thr by ProcM[4]. The Ser residue in PEP331, which is also located in between the lanthionine rings, escapes dehydration and is not phosphorylated. PEP332, PEP334 and PEP335 have a dehydrobutyrine in between the lanthionine rings demonstrating that dehydration is sequence and structure dependent. In blue the b- and y-ions are shown from Supplementary Tables 10 – 15. The thioether configuration in PEP333 could not be unambiguously identified and is currently under further investigation.

Supplementary Figure 6

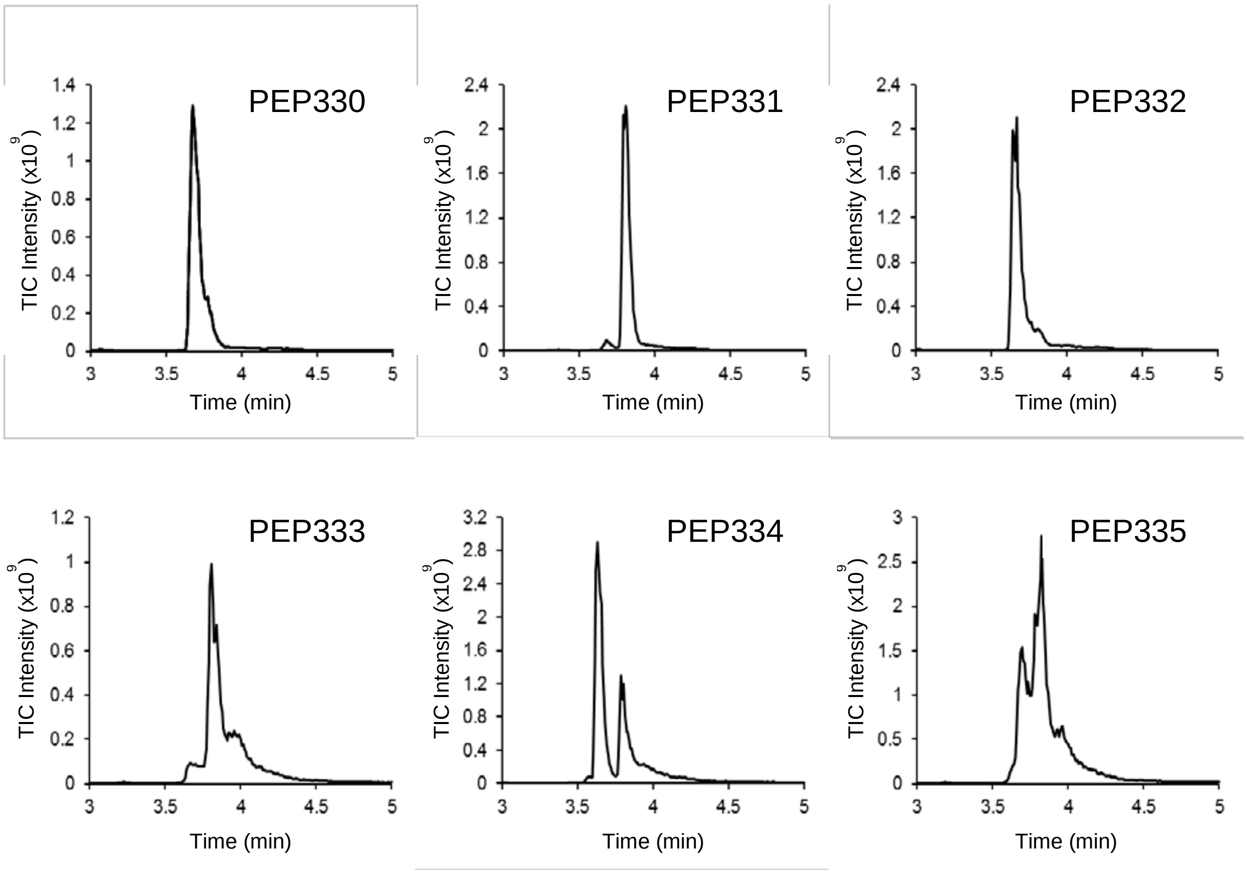

Supplementary Figure 6 UPLC profiles of phage-selected lanthipeptides analyzed as leader-core peptides with C-terminal His_6-tag. The data were collected by using a gradient from 5 to 95% acetonitrile in water with 0.1% formic acid over 10 min at a flow rate of 0.3 ml min^{-1} on an Acquity UPLC Protein BEH C18 column (2.1 mm x 50 mm, Waters). The time course of elution was detected with Total Ion Current (TIC) Intensity.

Supplementary Figure 7

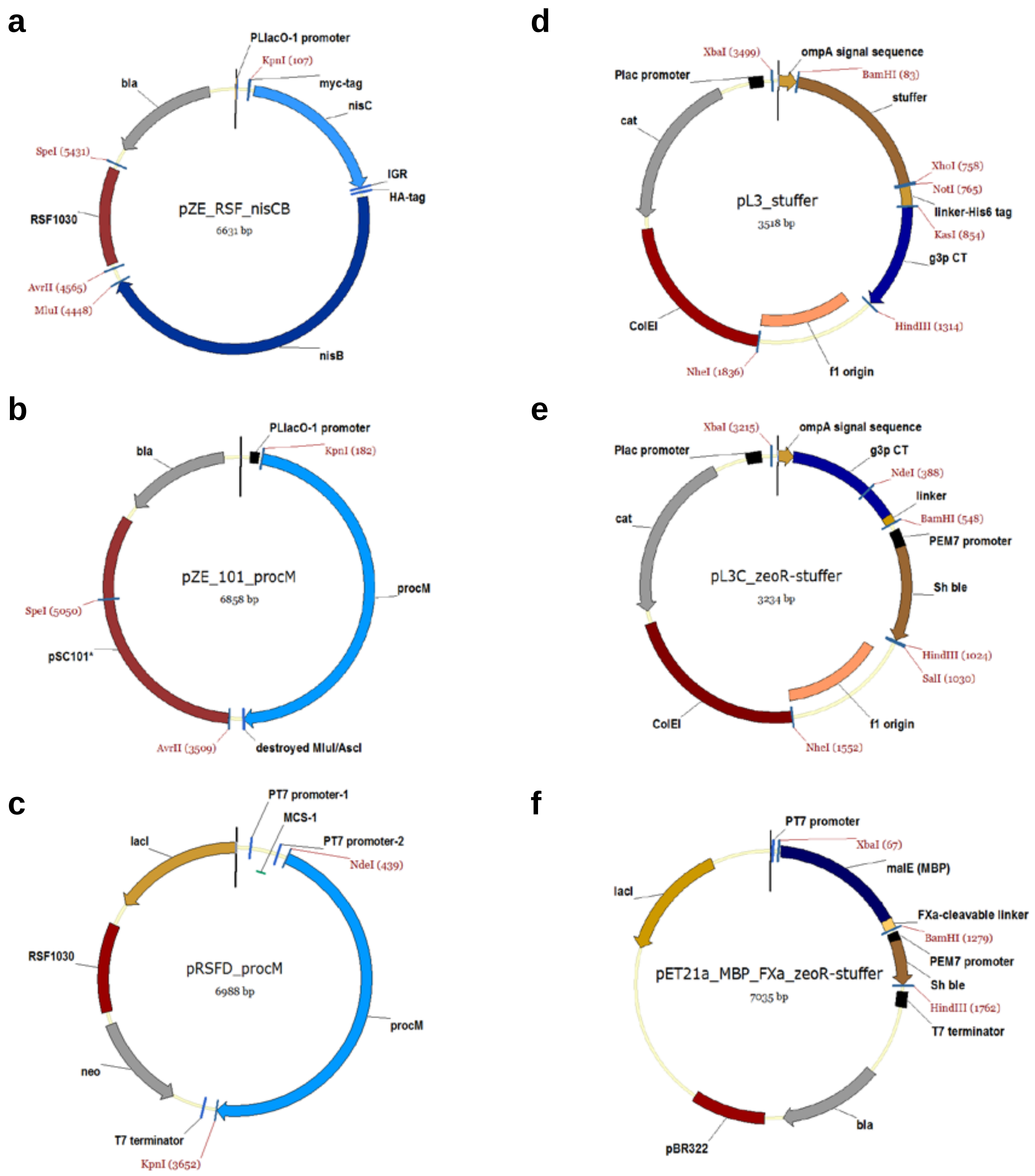

Supplementary Figure 7 Vector maps of plasmids used in this study. (**a**) pZE_RSF_nisCB: Expression of a bicistronic operon encoding *L. lactis nisC* and *nisB* genes with N-terminal *myc*-, and HA-tags, respectively, from an inducible PLlacO-1 promoter. (**b**) pZE_101_procM: Expression of *Prochlorococcus* MIT9313 *procM* from an inducible PLlacO-1 promoter. (**c**) pRSF_procM: Expression of *Prochlorococcus* MIT9313 *procM* from an inducible T7 promoter. (**d**) pL3_stuffer: Plasmid/phagemid for expression of peptide fusions to the N-terminus of pIII from an inducible Plac promoter and with an f1 origin for single strand DNA production. (**e**) pL3C_zeoR-stuffer: Plasmid/phagemid with same features as (d), but for expression of peptide fusions to the C-terminus of pIII. (**f**) pET21a_MBP_FXa_zeoR-stuffer: Expression of peptide fusions to the C-terminus of *malE* (MBP) via an FXa-cleavable linker from an inducible T7 promoter. Genes: *bla*, *neo*, *cat* conferring ampicillin, kanamycin, and chloramphenicol resistance, respectively. *lacI*: lac repressor. Replicons: RSF1030 (100 copies/cell), ColE1 (~ 20 copies/cell), pBR322 (~ 20 copies/cell), pSC101* (3-4 copies/cell).

Supplementary Table 1

**Amino acid sequence of NisA-leader containing model lanthi-precursor peptides expressed from pL3_stuffer derivatives**	
OmpA(1-21)::GGGGSAHKHKHKPGGSGGAPGSMSTKDFNLDLVSVSKKDSGASPR::XXX[a]	
pL_Nis-6[b]	PAGDYKDDDDKA**S**W**IEGR**E**C**NAAAGPHHHHHH[c]
pL_Nis-6-S36T[b]	PAGDYKDDDDKA**T**W**IEGR**E**C**NAAAGPHHHHHH[c]
pL_Nis-6-S36A[b]	PAGDYKDDDDKA**A**W**IEGR**E**C**NAAAGPHHHHHH[c]
pL_Nis-6-C43A[b]	PAGDYKDDDDKA**S**W**IEGR**E**A**NAAAGPHHHHHH[c]

[a] The N-terminal sequence shared by all constructs is comprised of the OmpA signal sequence (OmpA residues 1-21) fused to a linker sequence containing an (HK)$_3$-repeat for improved expression and the natural NisA-leader sequence (underlined); XXX indicates core peptide sequences as shown in the individual rows below.
[b] Plasmid trivial name used in this study.
[c] Core peptide sequence with FLAG- and His$_6$-tags used for ELISA capture/detection (shaded), FXa-site (bold, underlined), and relevant residues for thioether-bridge formation (double-underlined).

Supplementary Table 2

**Amino acid sequence of ProcA-leader containing model lanthi-precursor peptides expressed from pL3_stuffer derivatives**	
OmpA(1-21)::<br>GGGGSAHKHKHKPGGSGGAPGSMSEEQLKAFIAKVQGDSSLQEQLKAEGADVVAIAKAAGFTIKQQDLNAAASELSDEELEAASGG::XXX[a]	
pL_Proc-6[b]	PAGYPYDVPDYA**S**W**IEGR**E**C**NAAAGPHHHHHH[c]
pL_Proc-6-S77T[b]	PAGYPYDVPDYA**T**W**IEGR**E**C**NAAAGPHHHHHH[c]
pL_Proc-6-S77A[b]	PAGYPYDVPDYA**A**W**IEGR**E**C**NAAAGPHHHHHH[c]
pL_Proc-6-C84A[b]	PAGYPYDVPDYA**S**W**IEGR**E**A**NAAAGPHHHHHH[c]

[a] The N-terminal sequence shared by all constructs is comprised of the OmpA signal sequence (OmpA residues 1-21) fused to a linker sequence containing an (HK)$_3$-repeat for improved expression and the natural ProcA3.3-leader sequence (underlined); XXX indicates core peptide sequences as shown in the individual rows below.
[b] Plasmid trivial name used in this study.
[c] Core peptide sequence with HA- and His$_6$-tags used for ELISA capture/detection (shaded), FXa-site (bold, underlined), and relevant residues for thioether-bridge formation (double-underlined).

Supplementary Table 3

Amino acid sequence of NisA-leader containing model lanthi-precursor peptides displayed on phage pIII CT and expressed from pL3_stuffer, or pL3C_zeoR-stuffer derivatives	
OmpA(1-21)::GGGGSAGSMSTKDFNLDLVSVSKKDSGASPR::XXX::GGGDSRGGGAAGGGDSRGGGA::pIII CT (275-424)[a]	
pL3_Nis-6[c]	PAGDYKDDDDKASWIEGRECNAAAGPHHHHHH[d]
OmpA(1-21)::pIII CT (275-424):: YSSAETDRSAPMSTKDFNLDLVSVSKKDSGASPR::XXX[b]	
pL3C_Nis-6[c]	PAGDYKDDDDKASWIEGRECNAAAGPHHHHHH[d]
pL3C_Nis-6-S36A[c]	PAGDYKDDDDKAAWIEGRECNAAAGPHHHHHH[d]
pL3C_Nis-6-C43A[c]	PAGDYKDDDDKASWIEGREANAAAGPHHHHHH[d]
pL3C_Nis-9[c]	PAGDYKDDDDKASWAAIEGRAECNAAAGPHHHHHH[d]
pL3C_Nis-12[c]	PAGDYKDDDDKASWAAAIEGRAAAECNAAAGPHHHHHH[d]
pL3C_Nis-16[c]	PAGDYKDDDDKASWAAGAAIEGRAAGAAECNAAAGPHHHHHH[d]

[a] Model lanthi-precursor peptide fusion to the N-terminus of pIII is comprised of the OmpA signal sequence (OmpA residues 1-21), a short linker, the NisA-leader (underlined), the core sequence (indicated by XXX; sequence in the row below), a linker, and residues 275-424 of phage pIII.
[b] The sequence shared by all lanthi-precursor peptides fused to the C-terminus of pIII is comprised of the OmpA signal sequence (OmpA residues 1-21), residues 275-424 of phage pIII, a linker sequence, and the NisA-leader (underlined); XXX indicates core peptide sequences as shown in the individual rows below.
[c] Plasmid trivial name used in this study.
[d] Core peptide sequence with FLAG- and His_6-tags (shaded), FXa-site (bold, underlined), and relevant residues for thioether-bridge formation (double-underlined).

Supplementary Table 4

Amino acid sequence of ProcA-leader containing model lanthi-precursor peptides displayed on phage pIII CT and expressed from pL3_stuffer, or of pL3C_zeoR-stuffer derivatives	
OmpA(1-21)::GGGGSAGSMSEEQLKAFIAKVQGDSSLQEQLKAEGADVVAIAKAAGFTIKQQDLNAAASELSDEELEAASGG::XXX::GGGDSRGGGAAGGGDSRGGGA::pIII CT (275-424)[a]	
pL3_Proc-6[c]	PAGYPYDVPDYASWIEGRECNAAAGPHHHHHH
OmpA(1-21):: pIII CT (275-424):: YSSAETDRSAPGSMSEEQLKAFIAKVQGDSSLQEQLKAEGADVVAIAKAAGFTIKQQDLNAAASELSDEELEAASGG::XXX[b]	
pL3C_Proc-6[c]	PAGYPYDVPDYASWIEGRECNAAAGPHHHHHH[d]
pL3C_Proc-6-S77A[c]	PAGYPYDVPDYAAWIEGRECNAAAGPHHHHHH[d]
pL3C_Proc-6-C84A[c]	PAGYPYDVPDYASWIEGREANAAAGPHHHHHH[d]
pL3C_Proc-9[c]	PAGYPYDVPDYASWAAIEGRAECNAAAGPHHHHHH[d]
pL3C_Proc-12[c]	PAGYPYDVPDYASWAAAIEGRAAAECNAAAGPHHHHHH[d]
pL3C_Proc_16[c]	PAGYPYDVPDYASWAAGAAIEGRAAGAAECNAAAGPHHHHHH[d]

[a] Model lanthi-precursor peptide fusion to the N-terminus of pIII is comprised of the OmpA signal sequence (OmpA residues 1-21), a short linker, the ProcA3.3-leader (underlined), the core sequence (indicated by XXX; sequence in the row below), a linker, and residues 275-424 of phage pIII.
[b] The sequence shared by all lanthi-precursor peptides fused to the C-terminus of pIII is comprised of the OmpA signal sequence (OmpA residues 1-21), residues 275-424 of phage pIII, a linker sequence, and the ProcA3.3-leader (underlined); XXX indicates core peptide sequences as shown in the individual rows below.
[c] Plasmid trivial name used in this study.
[d] Core peptide sequence with HA- and His_6-tags (shaded), FXa-site (bold, underlined), and relevant residues for thioether-bridge formation (double-underlined).

Supplementary Table 5

**Amino acid sequence of ProcA-leader containing lanthi-precursor peptides fused to the C-terminus of MBP and expressed from pET21a_MBP_FXa_zeoR-stuffer derivatives**	
MBP(Met,27-391)NSSSNNNNNNNNNNGT**IEGR**GSPGGSGGAPGS::<br>MSEEQLKAFIAKVQGDSSLQEQLKAEGADVVAIAKAAGFTIKQQDLNAAASELSDEELEAASGG::XXX[a]	
pET21a_MBP_ProcA1.1[b]	FFCVQGTANRFTINVCAAAGPHHHHHH[c]
pET21a_MBP_ProcA2.8[b]	AACHNHAPSMPPSYWEGECAAAGPHHHHHH[c]
pET21a_MBP_ProcA2.11[b]	GRIDTCPAGGGTSEQTGTCCAAAGPHHHHHH[c]
pET21a_MBP_PEP226[b]	**IEGR**GAACHNHAPSMPPSYWEGECAAGPHHHHHH[c]
pET21a_MBP_PEP330[b]	TNCGRKMSATYSGQKMYCIAAGPHHHHHH[c]
pET21a_MBP_PEP331[b]	ANCIQHLTHSPTVHKYFCPAAGPHHHHHH[c]
pET21a_MBP_PEP332[b]	AFCMRHHSTRTSENRRDCQAAGPHHHHHH[c]
pET21a_MBP_PEP333[b]	AICQIADATRTGENRKCLAAGPHHHHHH[c]
pET21a_MBP_PEP334[b]	ANCKVRQSWTRTYENKKCPAAGPHHHHHH[c]
pET21a_MBP_PEP335[b]	AWCKWNMTKTKTYENVQCKAAGPHHHHHH[c]
**Amino acid sequence of antimicrobial lantibiotic precursor peptides fused to the C-terminus of MBP and expressed from pET21a_MBP_FXa_zeoR-stuffer derivatives**	
MBP(Met,27-391)NSSSNNNNNNNNNNGT**IEGR**GSPGGSGGAPGS::XXX[d]	
pET21a_MBP_His_6_ProcA3.3-LctA[b]	HHHHHHGTDPNSMSEEQLKAFIAKVQGDSSLQEQLKAEGADVVAIAKAAGFTIKQQDLN<br>AAASELSDEELEAASGG**KGGSGVIHTISHECNMNSWQFVFTCCS**[e]
pET21a_MBP_His_6_NisA[b]	HHHHHHGTMSTKDFNLDLVSVSKKDSGASPR**ITSISLCTPGCKTGALMGCNMKTATCHC**<br>**SIHVSK**[e]

[a] The N-terminal sequence shared by all constructs, with the exception of PEP226, is comprised of MBP residues 27-391 (Met start codon, no signal sequence) fused to a linker sequence containing an internal FXa-site, and the natural ProcA3.3-leader sequence (underlined); in PEP226 the linker sequence was replaced by NSSSNNNNNNNNNNGTPGGSGGAPGS to eliminate the FXa-site; XXX indicates core peptide sequences as shown in the individual rows below.
[b] Plasmid trivial name used in this study.
[c] Core peptide sequence with His_6-tag (shaded).
[d] The N-terminal sequence shared by the two lantibiotic fusions is comprised of MBP residues 27-391 (Met start codon, no signal sequence) fused to a linker sequence containing an internal FXa-site; XXX indicates peptide precursor sequences as shown in the individual rows below.
[e] Precursor sequences consisting of the ProcA3.3 and NisA leader sequences (underlined), the LctA and NisA core peptides (bold), and N-terminal His_6-tag (shaded).

Supplementary Table 6

Construct	Sequence[a]	Sequence mass[b] (g/mol)	No of dehydr. found (possible)	Mass[b] fully dehydrated (g/mol)	Mass[b] found (g/mol)
**Sequence and mass of prochlorosin lanthipeptides produced as C-terminal MBP fusions**					
PEP223	GSPGGSGGAPGSMSEEQLKAFIAKVQGDSSLQEQLK AEGADVVAIAKAAGFTIKQQDLNAAASELSDEELEA ASGG**FFCVQGTANRFTINVC**AAAGPHHHHHH	10470.4	2 (2)	10434.3	10434.3
PEP224	GSPGGSGGAPGSMSEEQLKAFIAKVQGDSSLQEQLK AEGADVVAIAKAAGFTIKQQDLNAAASELSDEELEA ASGG**AACHNHAPSMPPSYWEGEC**AAAGPHHHHHH	10737.6	2 (2)	10701.5	10701.5
PEP225	GSPGGSGGAPGSMSEEQLKAFIAKVQGDSSLQEQLK AEGADVVAIAKAAGFTIKQQDLNAAASELSDEELEA ASGG**GRIDTCPAGGGTSEQTGTCC**AAAGPHHHHHH	10564.3	5 (5)	10474.3	10474.3
PEP226	**GAACHNHAPSMPPSYWEGEC**AAGPHHHHHH	3261.3[c]	2 (2)	3225.3[c]	3225.3[c]

[a] The sequence of the ProcA3.3-leader (underlined), the core peptide (bold), and putative sites of dehydration (double underlined) are highlighted
[b] For peptide > 10 kDa the average mass is shown
[c] For PEP226 (< 10 kDa) the monoisotopic mass is shown

Supplementary Table 7

Fragments of c-series identified in mass spectra of peptide ProcA2.8 (PEP226) under ETD conditions

		c_n^{1+}		c_n^{2+}		c_n^{3+}	
Residue[a]	Fragment	m/z calc.[b]	m/z found	m/z calc.[b]	m/z found	m/z calc.[b]	m/z found
G	c_{1}	75.06		38.03		25.69	
A	c_{2}	146.09	146.13	73.55		49.36	
A	c_{3}	217.13	217.15	109.07		73.04	
C	c_{4}	320.14		160.57		107.38	
H	c_{5}	457.20		229.10		153.07	
N	c_{6}	571.24		286.12		191.09	
H	c_{7}	708.30		354.65		236.77	
A	c_{8}						
P	c_{9}	876.39		438.70		292.80	
Dha (S)	c_{10}	945.41	945.44	473.21	473.23	315.80	
M	c_{11}						
P	c_{12}						
P	c_{13}	1270.56	1270.56	635.78	635.80	424.19	
Dha (S)	c_{14}	1339.58		670.29		447.20	
Y	c_{15}	1484.63		742.82		495.55	
W	c_{16}	1652.70		826.85		551.57	
E	c_{17}	1763.73		882.37		588.58	
G	c_{18}	1802.74		901.88		601.59	
E	c_{19}	1913.78		957.39		638.60	
C	c_{20}	2106.84		1053.92		702.95	702.96
A	c_{21}	2177.87		1089.44	1089.47	726.62	726.65
A	c_{22}	2248.91		1124.96	1125.00	750.30	750.33
G	c_{23}						
P	c_{24}	2402.99		1201.99	1202.03	801.66	801.69
H	c_{25}	2540.04		1270.52	1270.56	847.35	867.38
H	c_{26}	2677.10		1339.05	1339.09	893.03	893.07
H	c_{27}	2814.16		1407.58	1407.62	938.72	938.76
H	c_{28}	2951.22		1476.11	1476.17	984.41	984.45
H	c_{29}	3088.28		1544.64	1544.70	1030.09	1030.12
H	c_{30}	3225.34		1613.17		1075.78	

[a] Ser residues were assumed to be dehydrated (dehydroalanine, Dha) since intact mass measurements indicate double dehydration (-36 Da).
[b] Calculated mass of singly (c_n^{1+}), doubly (c_n^{2+}) and triply (c_n^{3+}) charged c-fragments. Xaa-Pro sites do not give rise to fragments under ETD conditions due to the pyrrolidine structure of the proline side chain.
Sequences comprising the proposed thioether ring are highlighted (gray).

Supplementary Table 8

Fragments of z-series identified in mass spectra of peptide ProcA2.8 (PEP226) under ETD conditions							
		z_n^{1+}		z_n^{2+}		z_n^{3+}	
**Residue**[a]	**Fragment**	**m/z calc.**[b]	**m/z found**	**m/z calc.**[b]	**m/z found**	**m/z calc.**[b]	**m/z found**
H	z_1	140.05	140.09	70.53		47.35	
H	z_2	277.11	277.14	139.05		93.03	
H	z_3	414.17		207.58		138.72	
H	z_4	551.23		276.11		184.41	
H	z_5	688.29	688.31	344.64	344.67	230.09	
H	z_6	825.35	825.38	413.17	413.19	275.78	
P	z_7						
G	z_8	979.42		490.21	490.24	327.14	
A	z_9	1050.46		525.73		350.82	
A	z_{10}	1121.49		561.25	561.28	374.49	
C	z_{11}	1224.50		612.76		408.84	
E	z_{12}	1353.55		677.28		451.85	
G	z_{13}	1410.57		705.79		470.86	
E	z_{14}	1539.61		770.31		513.88	
W	z_{15}	1725.69		863.35		575.90	
Y	z_{16}	1888.75		944.88		630.26	
Dha (S)	z_{17}	1957.77	1957.81	979.39	979.43	653.25	653.30
P	z_{18}						
P	z_{19}						
M	z_{20}	2282.92	2282.99	1141.96	1142.00	761.64	761.70
Dha (S)	z_{21}	2351.94		1176.47		784.65	
P	z_{22}						
A	z_{23}	2520.03		1260.52		840.68	
H	z_{24}	2657.09		1329.05		886.37	
N	z_{25}	2771.13		1386.07		924.38	
H	z_{26}	2908.19		1454.60		970.07	
C	z_{27}	3011.20		1506.10		1004.40	
A	z_{28}	3082.24		1541.62		1028.08	
A	z_{29}	3153.28		1577.14	1577.18	1051.76	
G	z_{30}	3210.30		1605.65	1605.71	1070.76	1070.80

[a] Ser residues were assumed to be dehydrated (dehydroalanine, Dha) since intact mass measurements indicate double dehydration (-36 Da).

[b] Calculated mass of singly (z_n^{1+}), doubly (z_n^{2+}) and triply (z_n^{3+}) charged c-fragments. Xaa-Pro sites do not give rise to fragments under ETD conditions due to the pyrrolidine structure of the proline side chain.

Sequences comprising the proposed thioether ring are highlighted (gray).

Supplementary Table 9

Full length mass analysis of selected leader-core peptides released from MBP-fusion protein by Factor Xa digestion and chemically or enzymatically treated as indicated[a]

Peptide	Sequence[b]	Found m/z	Modification[c] (fraction %)[d]	Calc. m/z	SAP[f] found m/z	TCEP[g] found m/z	IAA[g] found m/z	IAA addition[h] (fraction %)[c]	Lanthionine rings
PEP330	**TNCGRKMSATYSGQKMYCIA**	10644.06	-4x H_2O (15%)	10644.07	10644.08	10644.07	10644.16	0	2
		10662.07	-3x H_2O (10%)	10662.08	10662.11	10662.05	10662.11	0	2
		10742.04	-3x H_2O + P (75%)	10742.04	---	10742.05	10742.07	0	2
PEP331	**ANCIQHLTHSPTVHKYFCPA**	10734.18	-2x H_2O (100%)	10734.18	n.d.	10734.18	10734.16	0	2
PEP332	**AFCMRHHSTRTSENRRDCQA**	10837.14	-4x H_2O (35%)	10837.15	n.d.	10837.15	10837.14	0	2
		10855.13	-3x H_2O (55%)	10855.16		10855.16	10855.15	0	2
		10935.12	-3x H_2O + P (10%)	10935.13	.	10935.13	10935.15	0	2
PEP333	**AICQIADATRTGENRKCLA**	10500.1	Mix of -2x H_2O	10501.12	n.d.	10501.13	10501.11	0 (15%)	2
			and -2x H_2O + SS	10499.10			10558.13	1 (65%)	1
							10615.09	2 (20%)	0
PEP333u[e]	**AICQIADATRTGENRKCLA**	10537.12	reduced	10537.14	n.d.	---	10651.18	2	0
PEP334	**ANCKVRQSWTRTYENKKCPA**	10832.26	-3x H_2O (70%)	10832.26	n.d.	10832.26	10832.25	0 (80%)	2
							10889.21	1 (20%)	1
		10928.21	-2x H_2O + P + SS (15%)	10928.22		10930.23	10986.23	1	0[i]
		11137.28	-3x H_2O + GSH + SS (15%)	11137.33		11139.35	11196.35	1	0[i]
PEP335	**AWCKWNMTKTKTYENVQCKA**	10882.22	-3x H_2O (100%)	10882.24	n.d.	10888.25	10882.24	0 (40%)	2
							10939.25	1 (60%)	1

[a] The monoisotopic mass [M+H]+ is shown.
[b]Peptide sequences are shown without the N-terminal ProcA3.3 leader (GSPGGSGGAPGSMSEEQLKAFIAKVQGDSSLQEQLKAEGADVVAIAKAAGFTIKQQDLNAAASELSDEE-LEAASGG) and the C-terminal His_6-tag (AGPHHHHHH). Putative sites of dehydration are underlined.
[c] Modifications found are –**nx H_2O**; dehydration of Ser and Thr (-H_2O; -18.01057 Da), **P**; phosphorylation (+HPO_3 ; +79.96633 Da), **SS**; disulfide formation (-2H; -2.01565 Da) and **GSH**; glutathione addition (+ 307.083801 Da).
[d] The height of the isotopic mass distribution was used to estimate the percentage of each fraction when multiple species are detected.
[e] PEP333u is peptide produced in absence of ProcM enzyme (u = unmodified) and was used as internal positive control for the iodoacetamide modification, since it has two free cysteine residues.
[f] SAP stands for treatment with shrimp alkaline phosphatase and only sample PEP330 was determined to demonstrate phosphorylation (n.d. is not determined).
[g] TCEP was used to reduce the peptide samples to demonstrate the presence of disulfide bridge.
[h] IAA stands for iodoacetamide modification of cysteine (+C_2H_3NO; + 57.02146 Da). The number of additions and their relative appearance based on isotopic mass distribution peak height is shown. Each reaction tube included PEP333u as a positive control for full IAA modification.
[i] Zero lanthionines is indicated here since these peptide species are disulfide-bridged and contain a free dehydroalanine, which reacts promptly with free cysteine at pH 8.

Supplementary Table 10

Fragments of b- and y-ions in mass spectra of peptide PEP332 under CID conditions[a]

b_n^{1+}		b_n^{2+}					y_n^{1+}		y_n^{2+}		$y_n\text{-}2NH_3^{2+}$ [b]	
**m/z calc.**	**m/z found**	**m/z calc.**	**m/z found**	**n**	**residue**	**n**	**m/z calc.**	**m/z found**	**m/z calc.**	**m/z found**	**m/z calc.**	**m/z found**
---		---		1	**A**	25	---		---		---	
143.082		---		2	**A**	24	2624.152		1312.580		1295.553	
230.113	230.114	---		3	**S**	23	2553.115		1277.061		1260.035	1260.039
287.135		---		4	**G**	22	2466.083		1233.545		1216.519	1216.523
344.156	344.156	---		5	**G**	21	2409.062		1205.035		1188.008	
415.194	415.194	---		6	**A**	20	2352.04		1176.524		1159.497	1159.494
562.262	562.267	---		7	**F**	19	2281.003		1141.005		1123.979	
665.271		---		8	**C**	18	2133.935		1067.471		1050.445	
796.312		---		9	**M**	17	2030.926		1015.967		998.940	
952.413		476.710		10	**R**	16	1899.885		950.446		933.420	
1089.472		545.239		11	**H**	15	1743.784		872.396		855.369	
1226.531		613.769		12	**H**	14	1606.725		803.866		786.840	
1295.552	1295.552[d]	648.280	648.283	13	**Dha**[c]	13	1469.666		735.337		718.310	
1378.589	1378.594	689.798		14	**Dhb**[c]	12	1400.645		700.826	700.827	683.800	683.800
1534.690		767.849	767.85	15	**R**	11	1317.608		659.307	659.310	642.281	
1635.738		818.373	818.373	16	**T**	10	1161.507	1161.513	581.257	581.257	564.230	
1704.759		852.883		17	**Dha**[c]	9	1060.459	1060.459	530.733		513.707	
1833.802		917.405		18	**E**	8	991.437		496.222		479.196	
1947.845		974.426		19	**N**	7	862.395		431.701		414.675	
2103.946		1052.477		20	**R**	6	748.352		374.680		357.653	
2260.047		1130.527		21	**R**	5	592.250		296.629		279.603	
2375.074		1188.041		22	**D**	4	436.149		---		---	
2478.083		1239.545		23	**C**	3	321.122		---		---	
2606.142		1303.575		24	**Q**	2	218.113		---		---	
---		---		25	**A**	1	90.055		---		---	

[a] CID (Collision-induced dissociation). The residues highlighted in gray are part of the proposed lanthionine ring.
[b] Due to the presence of 4 Arg residues in the sequence these masses can only be observed with loss of two ammonia.
[c] Dehydrated residue; Dhb (dehydrobutyrine), Dha (dehydroalanine).
[d] A very clear related c_{13}^{1+}-ion peak was detected (found m/z 1312.578 and calc. m/z 1312.579).

Supplementary Table 11

Fragments of b- and y-ions identified in mass spectra of peptide PEP334 under CID conditions[a]

b_n^{1+}		b_n^{2+}					y_n^{1+}		y_n^{2+}	
**m/z calc.**	**m/z found**	**m/z calc.**	**m/z found**	**n**	**residue**	**n**	**m/z calc.**	**m/z found**	**m/z calc.**	**m/z found**
---		---		1	**A**	24	---		---	
143.081		---		2	**A**	23	2530.219		1265.613	1265.621
230.113	230.113	---		3	**S**	22	2459.182		1230.095	1230.100
287.135	287.135	---		4	**G**	21	2372.150		1186.580	1186.577
344.156	344.156	---		5	**G**	20	2315.128		1158.068	1158.056
415.193	415.193	---		6	**A**	19	2258.107		1129.557	1129.546
529.236	529.239	---		7	**N**	18	2187.070		1094.039	1094.030
632.245		---		8	**C**	17	2073.027		1037.017	1037.004
760.340		380.674		9	**K**	16	1970.018		985.513	
859.409		430.208		10	**V**	15	1841.923		921.465	
1015.510		508.258		11	**R**	14	1742.854		871.931	
1143.568		572.28		12	**Q**	13	1586.753		793.880	
1212.590		606.798		13	**Dha**[b]	12	1458.695		729.851	
1398.669		699.838	699.839	14	**W**	11	1389.673	1389.661	695.340	
1481.706	1481.692	741.357	741.357	15	**Dhb**[b]	10	1203.594	1203.590	602.301	
1637.807		819.407	819.404	16	**R**	9	1120.556	1120.545	560.782	
1720.844		860.926		17	**Dhb**[a]	8	964.456	964.450	482.732	
1883.908		942.457		18	**Y**	7	881.419		441.213	
2012.950		1006.979		19	**E**	6	718.355		359.681	
2126.993		1064.001		20	**N**	5	589.313		295.160	
2255.088		1128.048		21	**K**	4	475.270		238.139	
2383.183		1192.096		22	**K**	3	347.175		174.091	
2486.192		1243.600		23	**C**	2	219.080		---	
---		---		24	**P**	1	116.071		---	

[a] CID (Collision-induced dissociation). The residues highlighted in gray are part of the proposed lanthionine ring.
[b] Dehydrated residue; Dhb (dehydrobutyrine), Dha (dehydroalanine).

Supplementary Table 12

Fragments of b- and y-ions identified in mass spectra of peptide PEP335 under CID conditions[a]

b_n^{1+}		b_n^{2+}					y_n^{1+}		y_n^{2+}	
**m/z calc.**	**m/z found**	**m/z calc.**	**m/z found**	**n**	**residue**	**n**	**m/z calc.**	**m/z found**	**m/z calc.**	**m/z found**
---		---		1	**A**	29	---		---	
143.082		---		2	**A**	28	3013.402		1507.205	
230.114		---		3	**S**	27	2942.365		1471.686	1471.684
287.135	287.135	---		4	**G**	26	2855.333		1428.170	1428.169
344.157	344.156	---		5	**G**	25	2798.311		1399.659	
415.194	415.193	---		6	**A**	24	2741.290		1371.149	1371.145
601.273	601.273	---		7	**W**	23	2670.253		1335.630	1335.626
704.282		---		8	**C**	22	2484.173		1242.590	1242.590
832.377		416.692		9	**K**	21	2381.164		1191.086	
1018.456		509.732		10	**W**	20	2253.069		1127.038	
1132.499		566.753		11	**N**	19	2066.990		1033.999	
1263.540		632.274		12	**M**	18	1952.947		976.977	
1346.577	1346.584	673.792		13	**Dhb[b]**	17	1821.907		911.4570	
1474.672	1474.681	737.840		14	**K**	16	1738.869	1738.869	869.938	
1557.709	1557.708	779.358	799.358	15	**Dhb[b]**	15	1610.774	1601.775	805.891	
1685.804	1685.803	843.406	843.406	16	**K**	14	1527.737	1527.737	764.372	
1768.841		884.924		17	**Dhb[b]**	13	1399.642	1399.643	700.325	
1931.904		966.456		18	**Y**	12	1316.605		658.806	
2060.947		1030.977		19	**E**	11	1153.542		577.275	
2174.990		1087.999		20	**N**	10	1024.499		512.753	
2274.058		1137.533		21	**V**	9	910.456		455.732	
2402.117		1201.562		22	**Q**	8	811.388		406.198	
2505.126		1253.067		23	**C**	7	683.329		342.168	
2633.220		1317.114		24	**K**	6	580.320	580.321	290.664	
2704.258		1352.633		25	**A**	5	452.225	452.225	226.616	
2775.295		1388.151		26	**A**	4	381.188	381.188	191.098	
2832.317		1416.662		27	**G**	3	310.151	310.151	155.579	
2929.370		1465.188		28	**P**	2	253.130	253.129	127.068	
---		---		29	**H**	1	156.077		78.542	

[a] CID (Collision-induced dissociation). The residues highlighted in gray are part of the proposed lanthionine ring.
[b] Dehydrated residue; Dhb (dehydrobutyrine), Dha (dehydroalanine).

Supplementary Table 13

Fragments of b- and y-ions identified in mass spectra of peptide PEP330 under CID conditions[a]

b_n^{1+}		b_n^{2+}					y_n^{1+}		y_n^{2+}	
**m/z calc.**	**m/z found**	**m/z calc.**	**m/z found**	**n**	**residue**	**n**	**m/z calc.**	**m/z found**	**m/z calc.**	**m/z found**
---		---		1	**A**	24	---		---	
143.082		---		2	**A**	23	2342.030		1171.519	1171.520
230.114		---		3	**S**	22	2270.993		1136.000	1136.000
287.135		---		4	**G**	21	2183.961		1092.484	1092.484
344.157	344.156	---		5	**G**	20	2126.939		1063.973	1063.976
427.194	427.190	---		6	**Dhb**[b,c]	19	2069.918		1035.463	1035.463
541.237	541.236	---		7	**N**	18	1986.881		993.944	993.943
644.246		---		8	**C**	17	1872.838		936.922	936.922
701.267		---		9	**G**	16	1769.828		885.418	
857.368		429.188		10	**R**	15	1712.807		856.907	
985.463		493.235		11	**K**	14	1556.706		778.857	
1116.50		558.756		12	**M**	13	1428.611		714.809	
1185.525	1185.512	593.266	593.266	13	**Dha**[b]	12	1297.57		649.289	
1256.562	1256.558	628.785		14	**A**	11	1228.549	1228.552	614.778	
1339.599		670.303		15	**Dhb**[b,c]	10	1157.512	1157.518	579.260	
1502.663		751.835		16	**Y**	9	1074.475	1074.475	537.741	
1571.684		786.346		17	**Dha**[b]	8	911.411		456.209	
1628.706		814.856		18	**G**	7	842.3900		421.699	
1756.764		878.886		19	**Q**	6	785.368		393.188	
1884.859		942.933		20	**K**	5	657.310		329.159	
2015.900		1008.454		21	**M**	4	529.215		---	
2178.963		1089.985		22	**Y**	3	398.174		---	
2281.972		1141.490		23	**C**	2	235.111		---	
---		---		24	**I**	1	132.102		---	

[a] CID (Collision-induced dissociation). The residues highlighted in gray are part of the proposed lanthionine ring.
[b] Dehydrated residue; Dhb (dehydrobutyrine), Dha (dehydroalanine).
[c] The triple charged phosphorylated species was used to generate these MS/MS data. Phosphorylation is not observed therefore one of these dehydrated residues results from MS/MS induced dephosphorylation.

Supplementary Table 14

Fragments of b- and y-ions identified in mass spectra of dephosphorylated[b] peptide PEP330 under CID conditions[a]

b_n^{1+}		b_n^{2+}					y_n^{1+}		y_n^{2+}	
**m/z calc.**	**m/z found**	**m/z calc.**	**m/z found**	**n**	**residue**	**n**	**m/z calc.**	**m/z found**	**m/z calc.**	**m/z found**
---		---		1	**A**	24	---		---	
143.081		---		2	**A**	23	2360.040		1180.523	1180.523
230.113	230.113	---		3	**S**	22	2289.003		1145.005	1145.005
287.135	287.119	---		4	**G**	21	2201.971		1101.489	1101.489
344.156	344.156	---		5	**G**	20	2144.949		1072.978	1072.975
427.194	427.193	---		6	**Dhb[b]**	19	2087.928		1044.467	1044.465
541.236	541.236	---		7	**N**	18	2004.891	2004.880	1002.949	1002.948
644.246		---		8	**C**	17	1890.848	1890.834	945.927	945.925
701.267		---		9	**G**	16	1787.839		894.423	
857.368		429.188		10	**R**	15	1730.817		865.912	
985.463		493.235		11	**K**	14	1574.716		787.862	
1116.504		558.755		12	**M**	13	1446.621		723.814	
1185.525	1185.521	593.266	593.264	13	**Dha[b]**	12	1315.581		658.294	
1256.562	1256.557	628.785	628.783	14	**A**	11	1246.559	1246.559	623.783	
1357.610		679.309		15	**T[c]**	10	1175.522	1175.521	588.265	
1520.673		760.840		16	**Y**	9	1074.474		537.741	
1589.695		795.351		17	**Dha[b]**	8	911.411		456.209	
1646.716		823.862		18	**G**	7	842.389		421.698	
1774.775		887.891		19	**Q**	6	785.368		393.1898	
1902.870		951.938		20	**K**	5	657.309		329.158	
2033.910		1017.459		21	**M**	4	529.214		---	
2196.974		1098.990		22	**Y**	3	398.174		---	
2299.983		1150.495	1150.508	23	**C**	2	235.111		---	
---		---		24	**I**	1	132.101		---	

[a] CID (Collision-induced dissociation). The residues highlighted in gray are part of the proposed lanthionine ring.
[b] Dehydrated residue; Dhb (dehydrobutyrine), Dha (dehydroalanine).
[c] Enzymatic dephosphorylation prior to MS/MS measurement results in a Thr residue instead of MS/MS breakdown product Dhb.

Supplementary Table 15

Fragments of b- and y-ions in mass spectra of peptide PEP331 under CID conditions[a]

b_n^{1+}		b_n^{2+}					y_n^{1+}		y_n^{2+}	
**m/z calc.**	**m/z found**	**m/z calc.**	**m/z found**	**n**	**residue**	**n**	**m/z calc.**	**m/z found**	**m/z calc.**	**m/z found**
---		---		1	**A**	29	---		---	
143.082		---		2	**A**	28	2865.346		1433.177	1433.178
230.114		---		3	**S**	27	2794.309		1397.658	1397.658
287.135	287.135	---		4	**G**	26	2707.277		1354.142	1354.142
344.156	344.156	---		5	**G**	25	2650.255		1325.631	1325.628
415.194	415.193	---		6	**A**	24	2593.234		1297.121	1297.119
529.236	529.236	---		7	**N**	23	2522.197		1261.602	1261.600
632.246		---		8	**C**	22	2408.154		1204.581	1204.578
745.330		---		9	**I**	21	2305.145		1153.076	
873.388		---		10	**Q**	20	2192.061		1096.534	
1010.447		505.727		11	**H**	19	2064.002		1032.505	
1123.531		562.269		12	**L**	18	1926.943		963.9752	
1206.568	1206.568	603.788		13	**Dhb**[b]	17	1813.859		907.433	
1343.627	1343.630	672.317	672.317	14	**H**	16	1730.822	1730.819	865.914	865.913
1430.659	1430.659	715.833	715.832	15	**S**	15	1593.763	1593.760	797.385	797.384
1527.712		764.360	764.386	16	**P**	14	1506.731	1506.730	753.869	753.868
1610.749		805.878		17	**Dhb**[b]	13	1409.678		705.343	705.342
1709.818		855.412		18	**V**	12	1326.641		663.824	
1846.877		923.942		19	**H**	11	1227.573		614.290	
1974.972		987.989		20	**K**	10	1090.514		545.760	
2138.035		1069.521		21	**Y**	9	962.419		481.713	
2285.103		1143.055		22	**F**	8	799.356		400.181	
2388.113		1194.560	1194.560	23	**C**	7	652.287		326.647	
2485.165		1243.086	1243.086	24	**P**	6	549.278	549.277	275.142	
2556.202		1278.605	1278.603	25	**A**	5	452.225	452.225	226.616	
2627.239		1314.123	1314.123	26	**A**	4	381.188	381.187	191.098	
2684.261		1342.634	1342.634	27	**G**	3	310.151	310.150	155.579	
2781.314		1391.161		28	**P**	2	253.130		127.068	
---		---		29	**H**	1	156.077		78.542	

[a] CID (Collision-induced dissociation). The residues highlighted in gray are part of the proposed lanthionine ring.
[b] Dehydrated residue; Dhb (dehydrobutyrine).

Supplementary Table 16:

List of primers used (in 5´ to 3´ direction)	
JUB-001	GTTTCCTAGGGTTATTGTCTCATGAGCGGATAC
JUB-002	GTTTACTAGTGTGCTACATTTGAAGAGATAAATTG
JUB-012	GTTTAACTTTAATAAGGAGATATACCATG
JUB-013	ATCTCCTTCTTAAAGTTAAACAAAGTTATTCTCATTTCCTCTTCCCTCC
JUB-014	CTTTGTTTAACTTTAAGAAGGAGATATACATATGGGCTATCCGTATGATGTGCCGGATTATGCGAG CGGCATAAAAAGTTCATTTAAAGCTCAACC
JUB-015	GTTTTACGCGTTCATTTCATGTATTCTTCCGAAAC
JUB-016	GTTTTGGTACCATGGGCGAACAGAAACTGATTAGCGAAGAAGATCTGAGCGGCGAAAGTCCAAATA AAAAAAATATAAAAAGAAATGTTGA
JUB-019	GTTTTGGCGCCGATTTTGATTATGAAAAAATGGC
JUB-020	GTTTTAAGCTTATCAAGACTCCTTATTACGCAGTATGTTAG
JUB-023	GCTAACATACTGCGTAATAAGGAG
JUB-024	GTTTTGGATCCCGCACTTCCACCGCCGCCGGCCTGCGCTACGGTAGC
JUB-007	GTTTTGGTACCATGGAAAGTCCATCATCTTGGAAAAC
JUB-009	GTTTTGGCGCGCCTTATTCAGTAGGCCAGAGACCAG
JUB-071	GTTTTGGTACCTTATTCAGTAGGCCAGAGACC
JUB-072	GTTTTCATATGGAAAGTCCATCATCTTGGAAAAC
JUB-136	GTTTTCCTAGGGTACGGGTTTTGC
JUB-137	GGTTTCTTAGACGTCAGGTG
JUB-225	Biotin~GCAGCGCAGAAACCGATCG
JUB-230	GTCGACGACCTGTGAAGTGAAAAATGG
JUB-231	AAGCTTTTATCAGTCCTGCTCCTCG
JUB-248	Biotin~CGTCGACAAGCTTTCATTAGTG

Supplementary Methods

Bioactivity assay

Sequences encoding the NisA precursor from *L. lactis* or a chimera consisting of the ProcA3.3 leader peptide fused to the LctA core peptide[1] were fused to the MBP gene via a linker that encodes an FXa cleavage site followed by a His_6-tag, resulting in plasmids pET21a_MBP_His_6_NisA and pET21a_MBP_His_6_ProcA-LctA, respectively. Heterologous production of MBP-fusions in *E. coli* and purification of leader-core peptides with N-terminal His_6-tag was performed as described above. In brief, pET21a_MBP_FXa_NisA was produced with and without co-expression of the NisBC enzymes, whereas pET21a_MBP_FXa_ProcA-LctA was produced with and without co-expression of the ProcM enzyme. Following purification of the MBP-fusions by Dextrin-Sepharose affinity chromatography and FXa-digest, the released His_6-tagged leader-core peptides were purified by IMAC and adjusted to a final concentration of 20 µM in PBS. Core peptides were released by treatment with 5 µM Trypsin (Sigma) for 3 h at 37°C followed by 10 h at 30°C and heat-inactivation at 99°C for 5 min. 40 µl of the resulting peptide solutions were directly spotted onto filter discs placed on solidified top-agar solutions containing appropriate indicator strain bacteria. Trypsin-treated PBS served as negative control. To assess the bioactivity of NisA (derived from pET21a_MBP_His_6_NisA production) a stationary phase *L. lactis* NZ9000 culture grown in GM17 media was diluted 1:25 into 15 ml GM17 top-agar and poured onto a 30 ml layer of bacteria-free GM17 agar. Authentic nisin was obtained from the cell-free supernatant of an *L. lactis* NZ9700 overnight culture grown in G17 media and served as positive control. To assess the bioactivity of the ProcA-LctA chimera (derived from pET21a_MBP_ His_6_ProcA-LctA production) a stationary phase *L. lactis* subsp. *cremonis* (HP) culture grown in M92 media was diluted 1:25 into 15 ml M92 top-agar and poured onto a 30 ml layer of bacteria-free M92 agar. Authentic lacticin 481 was obtained from the cell-free supernatant of an *L. lactis cremonis* CNRZ 481 overnight culture grown in M92 media and served as positive control.

Determination of inhibitory activity of lanthipeptides

The inhibitory activities of ProcM-modified and non-modified anti-uPA peptides were determined as previously described[2]. In brief, 1.5 nM human uPA (ProsPec, # enz-264-c; purified by size exclusion chromatography) were incubated with 50 µM of the fluorogenic substrate Z-Gly-Gly-Arg 7-amido-4-methylcoumarin hydrochloride (Santa Cruz Biotechnology, # sc-208012) and different concentrations of anti-uPA peptides. Substrate and peptides were diluted in 10 mM Tris-HCl (pH 7.4), 150 mM NaCl, 10 mM $MgCl_2$, 1 mM $CaCl_2$, 0.1% (w v^{-1}) BSA, 0.01% Triton X-100, 5% (v v^{-1}) DMSO buffer. The uPA activity was measured by monitoring the change in fluorescence intensity after an incubation step at 25°C for 30 minutes (black 96 well plate with clear flat bottom, Corning CellBIND, # 3340; excitation at 355 nm, emission recorded at 460 nm, Tecan M200pro, Thermo Scientific). A final inhibition constant K_i for PEP333 of 564 nM was calculated as previously reported[2].

Analysis of phage-selected peptides by mass spectrometry

Samples were analyzed by FTMS using a Shimadzu UFLC system (Shimadzu, Den Bosch, The Netherlands) coupled on-line via the HESI interface with a LTQ–Orbitrap-XL mass spectrometer (Thermo Fisher Scientific., San Jose, CA). Samples were loaded onto an Acquity UPLC, BEH C18. 50 x 2.1mm, 1.7µM (Waters, Ireland). The following mobile phase gradient was delivered at the flow rate of 0.3 ml min^{-1}: 5% solvent B for 1 min hold; linear gradient 5–95% solvent B in 10 min; 95% solvent B for 2 min. Solvent A was H_2O with 0.1% formic acid and solvent B was acetonitrile with 0.1% formic acid. The column temperature was kept constant at 60 °C. Typical spray voltage was 3 kV (heater 300 °C, sheath gas flow 40 and auxiliary gas flow 10 (arbitrary units); ion transfer tube temperature was 300 °C. Analysis consisted of full MS scans from m z^{-1} 380-2000 at the Orbitrap analyzer with target mass resolution of 100.000 (FWHM, full width at half maximum at m z^{-1} 400). The proportion of the full scan chromatogram corresponding to the peptide signal(s) was summed and mass spectra were extracted in Xcalibur v4.0 software before being deconvoluted using the Xtract

algorithm to give singly charged monoisotopic masses and the single charged isotopic patterns. HPLC-MS/MS analsysis of leader-free and His_6-tag free samples was performed using a Thermo/Dionex UltiMate UPLC coupled to a Thermo Q-Exactive mass spectrometer using the same gradient and column as mentioned above. Targeted MS/MS was predominantly performed on preselected triply charged ions at a resolution of at least 17500 @ m z^{-1} 200. Leader peptides were removed from peptides PEP330, PEP331, PEP332, PEP333, PEP334 and PEP335 by mild digestion with endoproteinase Glu-C (Sigma) in a 50 mM sodium phosphate buffer pH 7.6. The shortened peptides with a small part of the leader (AASGG) still attached were purified by HPLC on a Jupiter 4µ Proteo 90Å column 250 × 4.6 mm (Phenomonex) with a gradient of water and acetonitrile with both eluents containing 0.1% trifluoroacetate. The fraction containing the shortened peptide was dried in a vacuum concentrator. In order to obtain useful MS/MS data the linker-His_6-tag sequences were removed by using carboxypeptidase Y from (CPY) from Roche in a 50 mM Tris-HCl pH 7.4 buffer at 25 °C. Samples were 10 times diluted in 0.1% formic acid before analysis by HPLC-MS/MS. Iodoacetamide that alkylates free cysteine residues was used to demonstrate whether thioether rings have been formed. PEP333u, a peptide without modifications that was produced in the absence of the ProcM enzyme was included in each reaction tube. 20 µg of peptide and 10 µg of unmodified control PEP333u was dissolved in 100 µl of 100 mM ammonium bicarbonate buffer (ABC) pH 8.0 in the presence of 10 mM Tris(2-carboxyethyl) phosphine hydrochloride (TCEP-HCl) final concentration. The sample was incubated for 30 min at 37 °C and subsequently 5 µl of a freshly prepared 375 mM iodoacetamide (IAA) in 100 mM ABC was added to each sample and incubated for 30 min at room temperature in the dark. Samples were immediately diluted 10 times in 0.1% formic acid and analyzed by LC-MS. The full length and shortened peptide PEP330 was treated with shrimp alkaline phosphatase (SAP, Sigma) to demonstrate phosphorylation. 10 µg of peptide was dissolved in 100 µl of the supplied buffer and 5 units of SAP was added. The reaction was incubated at 37 °C for 3 hours. Samples were immediately diluted 10 times in 0.1% formic acid and analyzed by LC-MS.

Supplementary References

1. Zhang, Q., Yu, Y., Vélasquez, J.E. & van der Donk, Wilfred A. Evolution of lanthipeptide synthetases. *Proceedings of the National Academy of Sciences of the United States of America* **109,** 18361–18366 (2012).
2. Chen, S. *et al.* Bicyclic Peptide Ligands Pulled out of Cysteine-Rich Peptide Libraries. *J. Am. Chem. Soc.* **135,** 6562–6569 (2013).
3. Li, B. *et al.* Catalytic promiscuity in the biosynthesis of cyclic peptide secondary metabolites in planktonic marine cyanobacteria. *Proceedings of the National Academy of Sciences of the United States of America* **107,** 10430–10435 (2010).
4. Chatterjee, C. *et al.* Lacticin 481 synthetase phosphorylates its substrate during lantibiotic production. *Journal of the American Chemical Society* **127,** 15332–15333 (2005).
